# Association between omentin‐1 and heart failure with preserved ejection fraction in Chinese elderly patients

**DOI:** 10.1002/clc.24181

**Published:** 2023-11-08

**Authors:** Zhengjia Su, Shuya Tian, Wei Liang, Liqun Wu

**Affiliations:** ^1^ Department of Geriatrics, Ruijin Hospital Shanghai Jiao Tong University School of Medicine Shanghai China; ^2^ Department of Geriatrics, Shandong Provincial Third Hospital, Cheeloo College of Medicine Shandong University Shandon China; ^3^ Department of Cardiovascular Medicine, Ruijin Hospital Shanghai Jiao Tong University School of Medicine Shanghai China

**Keywords:** elderly patients, HFpEF, omentin‐1

## Abstract

**Background:**

Omentin‐1 is a novel adipokine and is associated with chronic inflammation and cardiovascular diseases. However, it remains unclear whether omentin‐1 levels are associated with diagnostic significance in elderly patients with heart failure with preserved ejection fraction (HFpEF). This study aimed to investigate the correlation between omentin‐1 and HFpEF in Chinese elderly patients.

**Hypothesis:**

Omentin‐1 may be invovled in HFpEF and there may be a difference of omentin‐1 levels between HFpEF and control.

**Methods:**

217 subjects were selected, including 115 patients with HFpEF and 102 control subjects. Enzyme‐linked immuno sorbent assay (ELISA) was used to detect plasma levels of omentin‐1, tumor necrosis factor‐α (TNF‐α) and interleukin‐6 (IL‐6). The receiver operating characteristics (ROC) curve was used to examine the diagnostic performance of omentin‐1 in HFpEF.

**Results:**

The levels of omentin‐1 decreased significantly in the HFpEF group (14.02 ± 8.35 vs. 19.74 ± 8.45 ng/mL, *p* < .001), while NT‐proBNP, IL‐6, and TNF‐α levels were significantly increased in the HFpEF group compared with the control group. Spearman correlation analysis showed that omentin‐1 levels were negatively correlated with E/e' (*r* = −.340, *p* < .001). The multivariate logistic regression analysis indicated that omentin‐1 was an independent protective factor for HFpEF (odd ratio = 0.948, 95% confidence interval [CI] 0.905–0.993, *p* = .025). Omentin‐1 levels were negatively correlated with NT‐proBNP (*r* = −.273, *p* < .001) and TNF‐α (*r* = −.221, *p* = .001). Diagnostic efficiency by ROC curve analysis in the patients with HFpEF showed that the area under the curve (AUC) for omentin‐1 was equivalent to NT‐proBNP (AUC: 0.734, 95%CI 0.667–0.802; AUC: 0.800, 95%CI 0.738–0.861). Subgroup analysis showed that in the patients between the age of 70 and 80, the predictive capability of omentin‐1 was stronger than NT‐proBNP (AUC: 0.809, 95%CI 0.680–0.937; AUC: 0.674, 95%CI 0.514–0.833).

**Conclusions:**

Omentin‐1 levels which were associated with inflammation, were decreased in the HFpEF patients. It could be regarded as a valuable biomarker for the occurrence and development of HFpEF in elderly patients.

## Graphical abstract

Omentin‐1 levels were significantly decreased in heart failure with preserved ejection fraction (HFpEF), and decreased omentin‐1 levels were associated with inflammation, diastolic dysfunction, and higher HFpEF risk. Omentin‐1 also appeared to predict HFpEF.

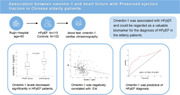

## 1 INTRODUCTION

Heart failure (HF), characterized by frequent hospitalization and reduced quality of life, accounts for the largest amount of mortality in the world. Approximately half of the HF patients have preserved left ventricular ejection fraction (LVEF), and HFpEF, which has been traditionally attributed to excessive left ventricular (LV) afterload, is the most prevalent form of HF in the elderly.^1^ However, there is a lack of completely understood clinical entity in HFpEF. Most epidemiological and clinical trials on HFpEF are based on imaging with echocardiography, whereas it is inconvenient for the elderly, especially for the disabled to go for ultrasound, and extensive plasma biomarkers have yet to be performed.^2^, ^3^

Many mechanisms have been proposed to clarify the development of HFpEF. Chronic inflammation is known to play a critical role. A lot of evidence demonstrated that inflammatory factors such as tumor necrosis factor‐α (TNF‐α) and interleukin‐6 (IL‐6) impair cardiac function, resulting in the enlargement of the ventricle, LV stiffening, depressed atrial compliance, and HF onset.^4^ Moreover, high levels of inflammatory mediators are associated with an unfavorable prognosis in HF.

Adipose tissue is able to produce pro‐inflammatory and anti‐inflammatory adipokines, which play an important role in cardiovascular disease. Omentin, which was discovered in 2003, is reported to have anti‐inflammatory, antioxidant, and antiapoptotic properties.^5^ Accumulating evidence indicates that omentin‐1 may be suitable candidate to influence cardiovascular diseases such as coronary artery disease, carotid atherosclerosis, hypertension, and ischemic HF.^6^ Up to now, studies investigating the relationship between omentin‐1 and HFpEF are still missing. It remains unclear whether omentin‐1 levels are associated with HFpEF in elderly patients. We hypothesized that omentin‐1 may be involved in HFpEF, and there may be a difference in omentin‐1 levels between HFpEF and control.

To this purpose, we measured the levels of plasma omentin‐1 in the old patients with HFpEF to clarify whether omentin‐1 could be a novel diagnostic biomarker of HFpEF in this study.

## 2 METHODS

### 2.1 Subject

Patients older than 60 years were recruited. Following the guidelines established by the European Society of Cardiology (ESC) in 2021,^7^ the diagnosis of HFpEF should include the following: (1) Symptoms and signs of HF (New York Heart Association Class II–IV). (2) LVEF ≥ 50%. (3) Objective evidence of cardiac structural and/or functional abnormalities consistent with the presence of LV diastolic dysfunction/raised LV filling pressures, including elevated levels of natriuretic peptides. The control group was defined as those who were without heart failure.

The exclusion criteria for all subjects were unstable clinical conditions (acute coronary syndrome, acute heart failure, acute cerebrovascular accident), inflammatory disease, history of cancer, severe chronic liver or renal disease, LV systolic dysfunction (EF < 50%), severe valvular heart disease, and myocardial hypertrophy.

A total of 217 subjects, comprising 115 patients with HFpEF and 102 control subjects, were recruited at Geriatric Department of Ruijin Hospital, affiliated to Shanghai Jiao Tong University School of Medicine from February 2018 to October 2019. Our study was approved by the Ruijin Hospital Ethics Committee and was conducted in accordance with the ethical principles stated in the Declaration of Helsinki.

### 2.2 Blood samples and enzyme‐linked immuno sorbent assay (ELISA)

Fasting blood samples were obtained the morning after admission. Blood samples were centrifuged at 3500 rpm at 4°C for 15 min. The plasma was stored at −80°C until further use. The concentrations of omentin‐1 and TNF‐α were detected using an ELISA kit (Shanghai Senxiong Bio‐Tech Co., Ltd.) following the manufacturer's instructions.

### 2.3 Statistical analysis

X ± s was used to stand for the measurement data with normal distribution. Frequency (*n*) or percentage (%) was used to represent the enumeration data. *T*‐test or Mann–Whitney *U* test was used for continuous variables. Multivariate logistic regression analysis was performed to evaluate the correlation between the independent variables and HFpEF. Spearman correlation analysis was used for the correlation test. The receiver operating characteristics (ROC) curve was plotted to assess the prognostic, predictive accuracy of different factors, and area under the curve (AUC) was estimated. *p* < .05 was regarded as a statistically significant difference.

## 3 RESULTS

### 3.1 Characteristics of study subjects

Characteristics of subjects with HFpEF (*n* = 115) and the corresponding age‐ and sex‐matched controls (*n* = 102) are described in Table 1. Compared with the control group, the HFpEF patients had greater values of NT‐proBNP, while there were no significant changes in age, blood pressure, fasting blood glucose (FGlu), total cholesterol (TC), triglyceride (TG), low‐density lipoprotein cholesterol (LDL‐c), very low‐density lipoprotein cholesterol (v‐LDL‐c), and high‐density lipoprotein cholesterol (HDL‐c) between the two groups (*p* > .05).

**Table 1:** Characteristics of HFpEF group and control group.

Indicator	HFpEF group (*n* = 115)	Control group (*n* = 102)	*p*‐Value
Age	76.87 ± 10.48	77.41 ± 11.23	.713
Gender			.080
Female	11	18	
Male	104	84	
History			
Hypertension (*n*,%)	91 (79.13%)	76 (74.51%)	.420
Diabetes (*n*,%)	57 (49.56%)	47 (46.08%)	.608
CHD (*n*,%)	46 (40.00%)	36 (35.29%)	.475
SBP (mmHg)	137.33 ± 20.20	137.52 ± 20.00	.970
DBP (mmHg)	71.45 ± 12.30	72.10 ± 10.90	.650
BMI (kg/m^2^)	24.75 ± 2.60	24.55 ± 2.60	.720
FGLU (mmol/L)	5.88 ± 1.27	6.03 ± 1.41	.423
HbA1C (%)	6.14 ± 1.01	6.11 ± 0.77	.816
TC (mmol/L)	4.37 ± 1.01	4.14 ± 0.97	.095
TG (mmol/L)	1.22 ± 0.06	1.12 ± 0.49	.380
LDL (mmol/L)	2.75 ± 1.00	2.50 ± 0.90	.054
HDL (mmol/L)	1.23 ± 0.39	1.24 ± 0.34	.878
vLDL (mmol/L)	0.66 ± 0.53	0.58 ± 0.16	.129
NT‐proBNP (pg/mL)	538.00 (395.10–972.00)	178.45 (73.00–468.70)	<.001
Echocardiographic characteristics			
EF (%)	64.90 ± 4.16	66.41 ± 2.95	.002
LADD (cm)	4.10 ± 0.40	4.00 ± 0.40	.088
LVPWT (cm)	0.90 ± 0.10	0.90 ± 0.10	.818
LVEDV (mL)	118.82 ± 17.84	115.19 ± 15.81	.116
LVESV (mL)	41.00 ± 10.16	38.17 ± 7.04	.019
E wave (cm/s)	69.72 ± 16.34	82.44 ± 21.86	<.001
A wave (cm/s)	92.39 ± 25.83	79.04 ± 26.96	<.001
E/A ratio	0.82 ± 0.38	1.24 ± 0.89	<.001
Septal e' wave(cm/s)	7.26 ± 1.87	10.06 ± 1.71	<.001
E/e' ratio	13.48 ± 4.02	10.25 ± 2.71	<.001

Abbreviations: A wave, peak late transmitral filling velocity; BMI, body mass index; CHD, coronary heart disease; DBP, diastolic blood pressure; e' wave, peak early diastolic mitral annulus velocity on tissue Doppler imaging; E wave, peak early transmitral filling velocity; FGLU, fasting blood glucose; HbA1C, glycosylated hemoglobin; HDL‐C, high‐density lipoprotein cholesterol; HFpEF, heart failure with preserved ejection fraction; LA, left atrial diameter; LDL‐C, low‐density lipoprotein cholesterol; LVPWT, left ventricular posterior wall thickness; LVEDV, left ventricular end‐diastolic volume; LVESV, left ventricular end‐systolic volume; SBP, systolic blood pressure; TC, total cholesterol; TG, triglyceride; vLDL‐C, very low‐density lipoprotein cholesterol.

As shown in Table 1, the HFpEF patients had larger left ventricular end‐systolic volume and lower left ventricular ejection fraction, although it was over 50%. In addition, E/e' ratio was significantly higher, while the septal e' wave and E/A ratio were significantly lower in the HFpEF group than in the control group (*p* < .001), indicating diastolic dysfunction.

### 3.2 Omentin‐1 levels decreased in HFpEF patients

**Figure 1:**
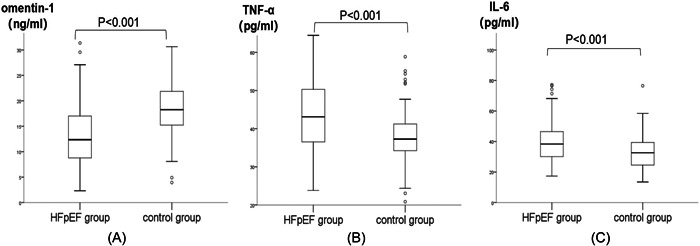
(A) Omentin‐1 levels decreased significantly in the HFpEF group. *p* < .001. (B) TNF‐α levels increased significantly in the HFpEF group. *p* < .001. (C) IL‐6 levels increased significantly in the HFpEF group. *p* < .001. HFpEF, heart failure with preserved ejection fraction; IL‐6, interleukin‐6; TNF‐α, tumor necrosis factor‐α.

The plasma omentin‐1 levels were greatly decreased in the HFpEF group compared with the control group (14.02 ± 8.35 ng/mL vs. 19.74 ± 8.45 ng/mL, *p* < .001, Figure 1A).

We additionally analyzed other inflammatory adipokines, including TNF‐α and IL‐6. TNF‐α and IL‐6 levels were significantly increased in the HFpEF group (44.62 ± 11.24 pg/mL vs. 38.70 ± 9.62 pg/mL, 39.77 ± 12.68 pg/mL vs. 33.41 ± 12.37 pg/mL, *p* < .001, Figure 1B,C).

### 3.3 Omentin‐1 reduced the risk of HFpEF

**Figure 2:**
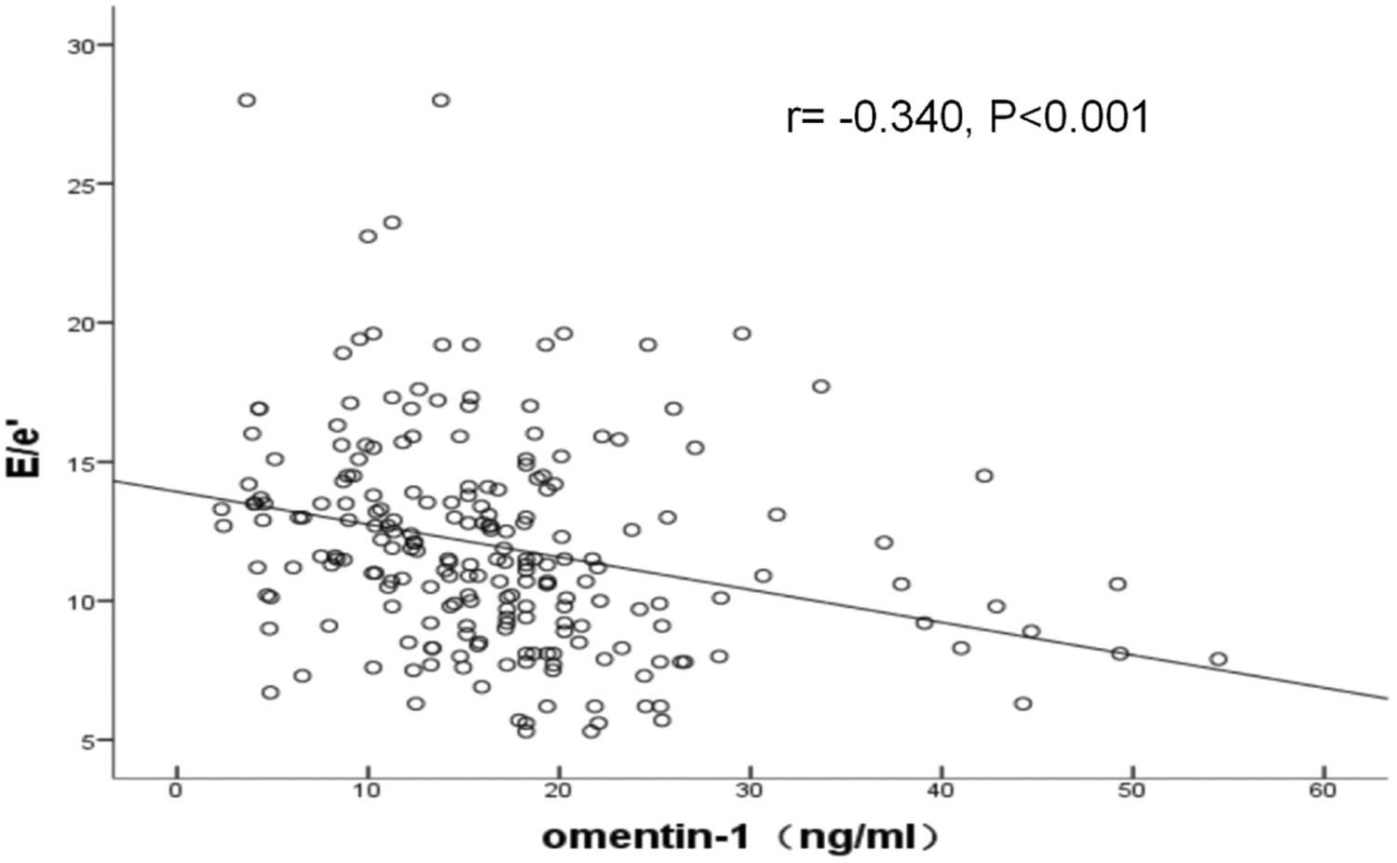
Omentin‐1 was negatively correlated with E/e' (*r* = −.340, *p* < .001).

Spearman correlation analysis showed that omentin‐1 levels were negatively correlated with E/e' (*r* = −.340, *p* < .001). (Figure 2) This indicated that the decreased level of omentin‐1 might be closely related with diastolic dysfunction.

**Figure 3:**
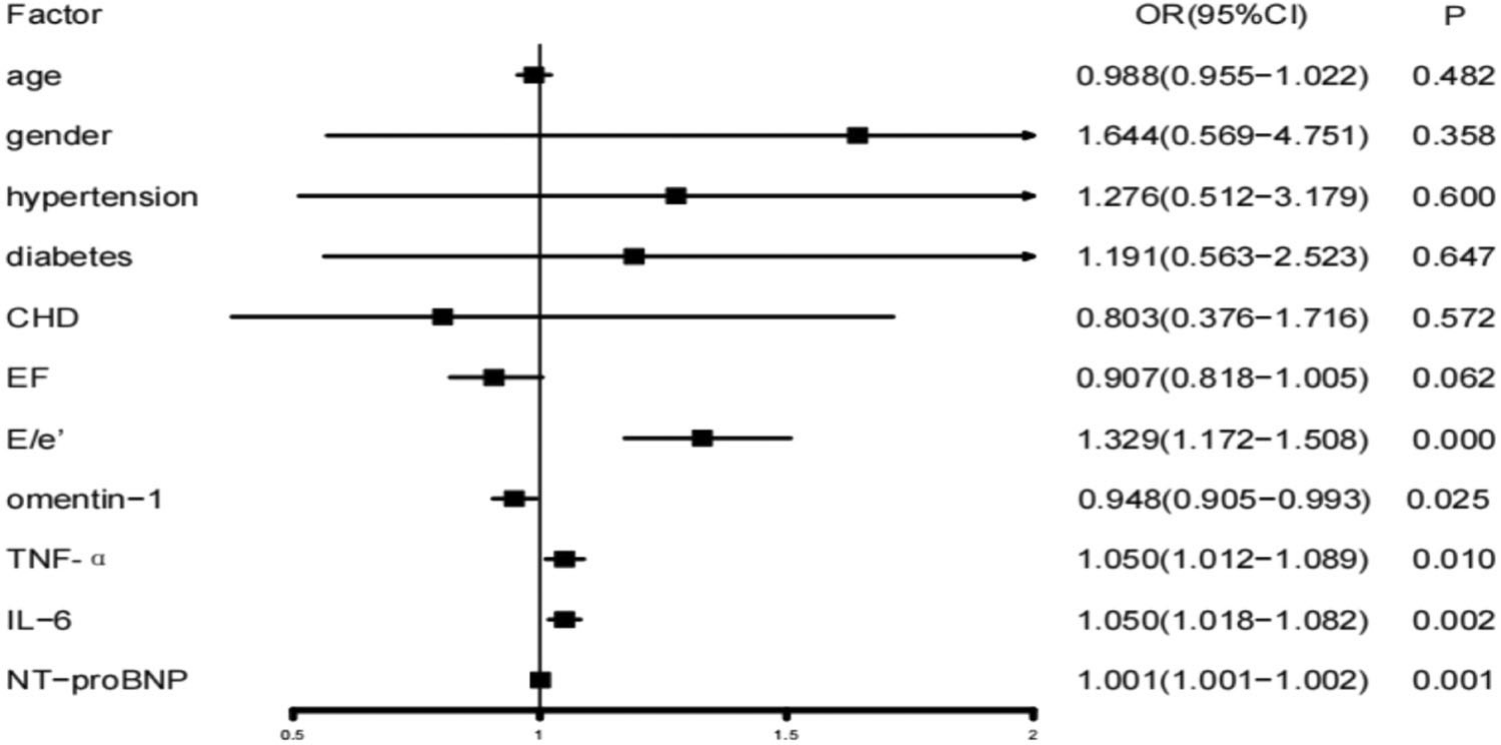
Multiple logistic regression analysis of factors influencing HFpEF. CI, confidence interval; HFpEF, heart failure with preserved ejection fraction; OR, odds ratio.

The multivariate logistic regression analysis demonstrated that the risk of HFpEF was significantly associated with omentin‐1, TNF‐α, IL‐6, and NT‐proBNP. A higher omentin‐1 level had a decreased risk for HFpEF (odds ratio = 0.948, 95% confidence interval [CI] 0.905–0.993, *p* = .025). Levels of TNF‐α, IL‐6, and NT‐proBNP could increase the risk of HFpEF (Figure 3).

The result indicated that omentin‐1 was an independent predictor of a decreased risk for HFpEF. However, TNF‐α, IL‐6, and NT‐proBNP were risk factors.

### 3.4 Analysis for the correlation between omentin‐1 and TNF‐α, IL‐6, NT‐proBNP

As omentin‐1 was found to decrease in HFpEF, while TNF‐α, IL‐6, and NT‐proBNP levels increased, the association among these inflammatory adipokines was further investigated.

A significant correlation between lower omentin‐1 levels and higher TNF‐α was observed (*r* = −.221, *p* = .001). We also found that omentin‐1 levels were negatively correlated with NT‐proBNP (*r* = −.273, *p* < .001). However, omentin‐1 was not found to be significantly associated with IL‐6 (*r* = .018, *p* = .796). (Supporting Information S1: Figure 1).

### 3.5 Association between omentin‐1 and HFpEF diagnosis

**Figure 4:**
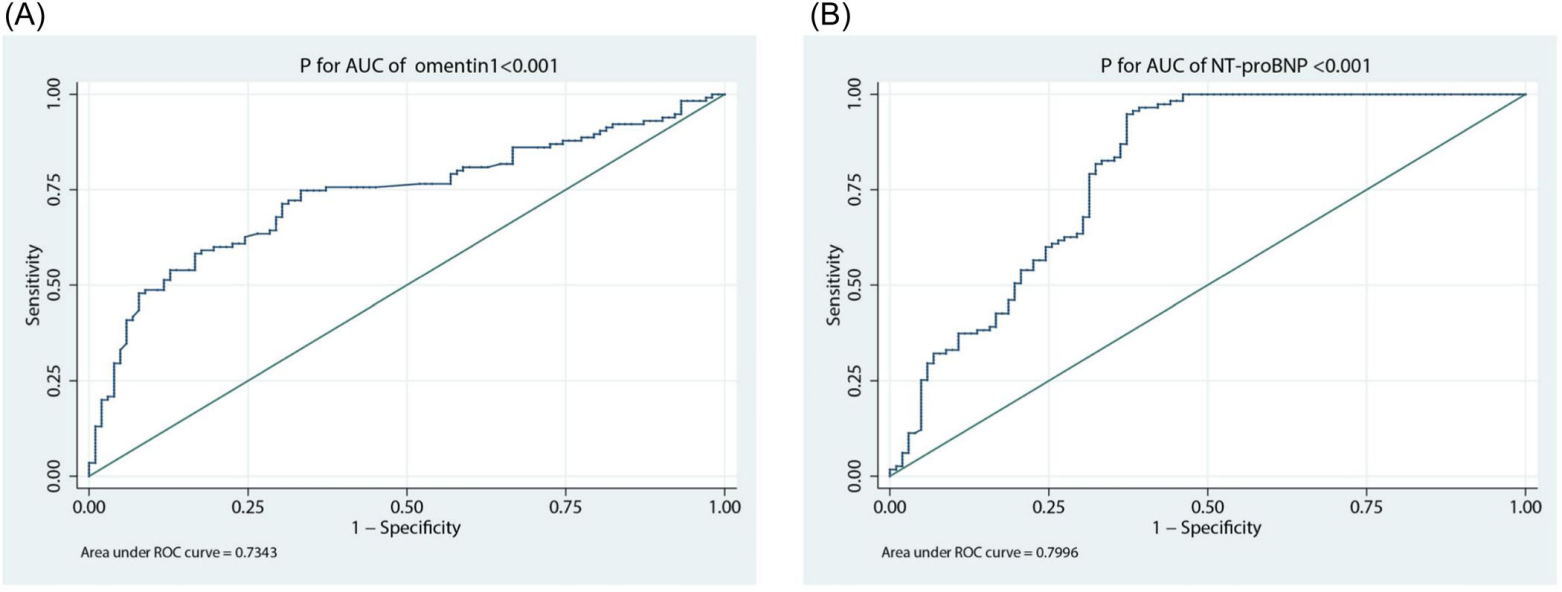
(A) In ROC curve analysis, omentin‐1 was predictive of HFpEF (AUC = 0.7343, 95% CI 0.667–0.802, *p* < .001). (B) The AUC for NT‐proBNP was 0.7996 in the patients with HFpEF (95% CI 0.738–0.861, *p* < .001). AUC, area under the curve; CI, confidence interval; HFpEF, heart failure with preserved ejection fraction; ROC, receiver operating characteristics.

ROC curve analysis showed that omentin‐1 was predictive of HFpEF (AUC = 0.734, 95% CI: 0.667–0.802). Its predictive capability was equivalent to that of NT‐proBNP (AUC = 0.800, 95% CI: 0.738–0.861) (Figure 4).

Furthermore, in our study, correlation analysis did not demonstrate a correlation between omentin‐1 and age (*r* = −.090, *p* = .188). Thus, we tried to split the subject into different age categories experimentally to analyze whether there was any difference in omentin‐1 impact. We referred to the age categories in other previous studies^8^ and divided the subjects into three groups: under the age of 70 (*n* = 66), between the age of 70 and 80 (*n* = 55) and patients over the age of 80 (*n* = 96).

Both omentin‐1 and NT‐proBNP performed well for HFpEF diagnosis in the group under 70 years old (AUC = 0.713, AUC = 0.816). Also, the predictive capability of these two markers was equivalent to that in the group over 80 years old (AUC = 0.705, AUC = 0.842). Interestingly, as a biomarker, omentin‐1 performed extremely better than NT‐proBNP for HFpEF diagnosis in the group between 70 and 80 years old (AUC = 0.809, AUC = 0.674) (Supporting Information S1: Figure 2).

## 4 DISCUSSION

Heart failure with preserved ejection fraction, lacking in compelling evidence base, represents a growing yet incompletely understood clinical entity. Most previous epidemiological and clinical trials on HFpEF were based on imaging with echocardiography.^9^ Recently, biomarkers are being increasingly used for the assessment of HFpEF, which has relevant impact on diagnosis and therapeutic strategies.

In the present study, we found that serum levels of omentin‐1 were lower in patients with HFpEF than in controls. A negative correlation was observed between serum levels of omentin‐1 and E/e', as well as NT‐proBNP and TNF‐α, in patients with HFpEF. Omentin‐1 might be a protective factor for HFpEF. ROC curve analysis also demonstrated that omentin‐1 might be predictive of HFpEF, in particular, its predictive capability was stronger than NT‐proBNP in the patients between 70 and 80 years old. Therefore, these results suggested that omentin‐1, a novel anti‐inflammatory adipokine, plays a role in HFpEF. To our knowledge, there is no study in the literature investigating the association between omentin‐1 and patients with HFpEF.

HFpEF, traditionally attributed to left ventricular diastolic dysfunction, has been reported as the induction of a systemic pro‐inflammatory state.^10^ A chronic pro‐inflammatory state, contributing to interstitial fibrosis, endothelial dysfunction, cardiac myocyte stiffening, and LV remodeling, appears to be the predominant pathophysiological mechanism in HFpEF.^11^, ^12^ Indeed, it has been demonstrated that increased inflammatory markers are associated with the development of HFpEF in elderly patients.^13^ Consistent with previous studies,^14^ TNF‐α and IL‐6, which are inflammatory biomarkers related to the pathogenesis of the disease, were found to be increased and relevant to a high risk of HFpEF in our study.

Omentin‐1, a new anti‐inflammatory cytokine released from visceral adipose tissue, not only participates in energy metabolism but also is associated with chronic inflammatory and autoimmune diseases.^15^ It was reported that increasing omentin‐1 concentrations may lower the risk of mortality among ischemic stroke patients.^16^ High omentin levels at hospital discharge could improve the prognosis of patients following acute heart failure.^17^ Furthermore, several studies have demonstrated that decreased levels of omentin‐1 may be involved in the occurrence and development of coronary artery disease and predict the prevalence of CHD.^18^, ^19^ In addition, the previous studies revealed that omentin levels were significantly decreased in HF and were closely associated with HF severity.^20^ In our study, the finding, supporting the perspective that omentin‐1 was a cardio‐protective adipokine showed that omentin‐1 levels were significantly decreased in HFpEF and reduced omentin‐1 might be associated with diastolic dysfunction and HFpEF risk. We also suggested that omentin‐1 could be a predictor of HFpEF diagnosis in the elderly. Even more importantly, NT‐proBNP, as a useful prognostic indicator in patients with HFpEF,^13^ was shown to be negatively correlated with omentin‐1 in our study. Nevertheless, we found that NT‐proBNP performed poorly for HFpEF diagnosis, while the predictive capability of omentin‐1 was better in patients between 70 and 80 years old. The concentration of NT‐proBNP is commonly influenced by comorbid conditions, consequently, some investigators have opined that an elevated natriuretic peptide concentration should no longer be required as a criterion for HFpEF.^21^, ^22^ Our findings indicated that measurement of omentin‐1 might be useful for HFpEF diagnosis in the elderly, in addition to NT‐proBNP.

A probable protective mechanism of omentin‐1 might be suppression of chronic inflammation and oxidative stress in patients with HFpEF. It was verified that omentin‐1 expression and production were decreased with elevated inflammatory adipokines, including TNF‐α and IL‐6, and omentin‐1 could prevent the TNF‐α‐induced superoxide or vascular inflammation.^23^, ^24^ Furthermore, omentin‐1 was reported to inhibit TNF‐α expression by blocking the ERK/NF‐κB pathway while ameliorate myocardial ischemic damage and apoptosis via blocking AMP‐activated protein kinase (AMPK) phosphorylation and Akt activity.^25^, ^26^ In agreement with the previous studies, we also found that omentin‐1 was negatively correlated with TNF‐α. Therefore, we speculate that omentin‐1 may contribute to TNF‐α inhibition that leads to slowing down the development of HFpEF or the prevention of the disease. However, the bioactivity of omentin‐1 in HFpEF appears multifaceted, and the exact mechanism remains to be investigated in the future.

In conclusion, we observed that omentin‐1 levels were significantly decreased in HFpEF, and decreased omentin‐1 levels were associated with inflammation, diastolic dysfunction, and higher HFpEF risk. More importantly, omentin‐1 appeared to predict HFpEF. Thus, omentin‐1 may serve as a novel prognostic marker for the occurrence and development of HFpEF. Intense research addressing the mechanism should be investigated in the future.

## CONFLICT OF INTEREST STATEMENT

The authors declare no conflict of interest.

## Supporting information

Supporting information.

Supporting information.

## Data Availability

The data that support the findings of this study are available from the corresponding author upon reasonable request.
