# Supplementary figures and images for: Association between omentin‐1 and heart failure with preserved ejection fraction in Chinese elderly patients
Source: Clin Cardiol. 2023 Nov 8;47(2):e24181. doi: 10.1002/clc.24181 (PMC10825884; doi:10.1002/clc.24181)

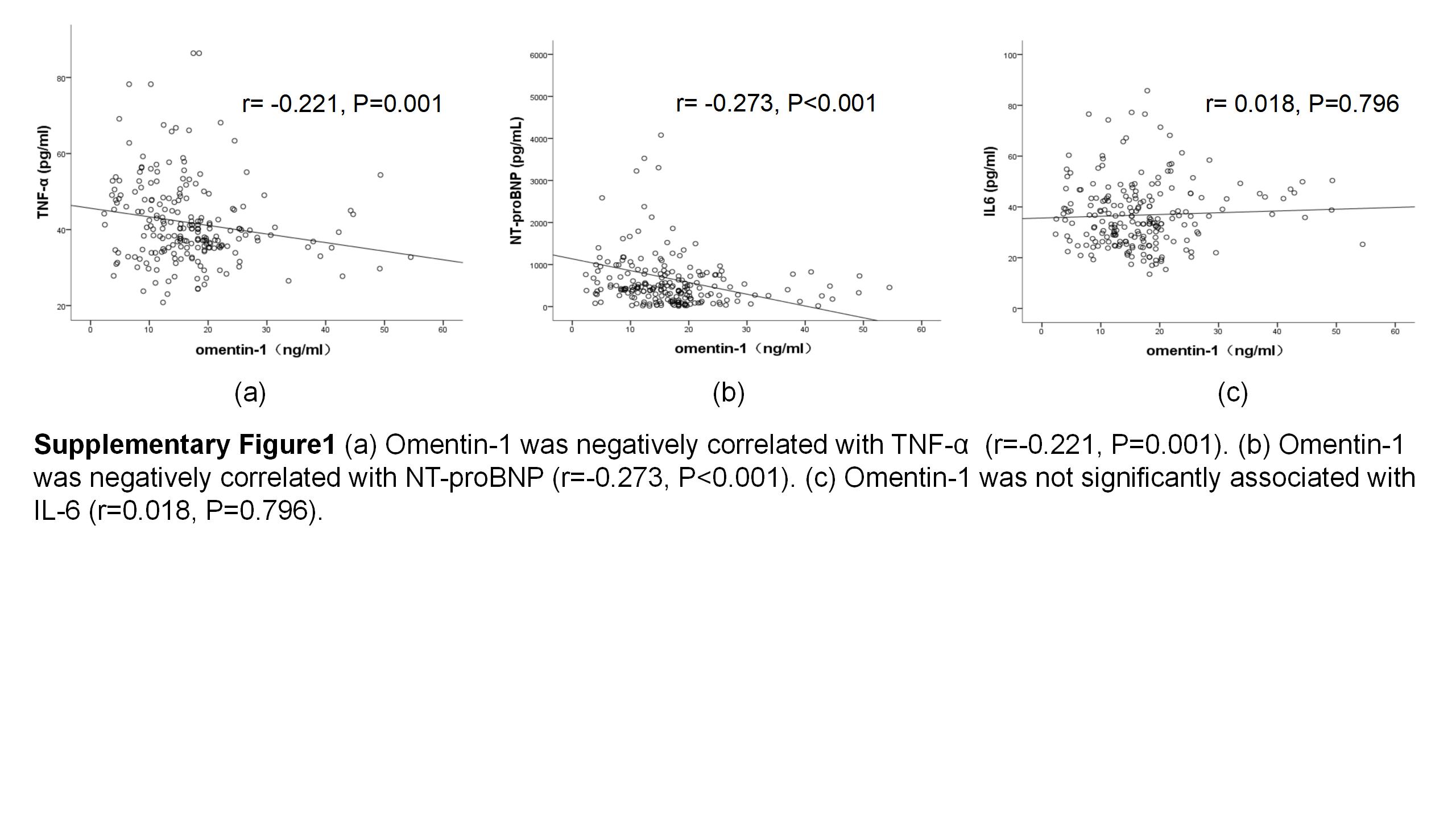

Supplement: Supplementary file 1 — Supporting information. [file CLC-47-e24181-s002.jpg]

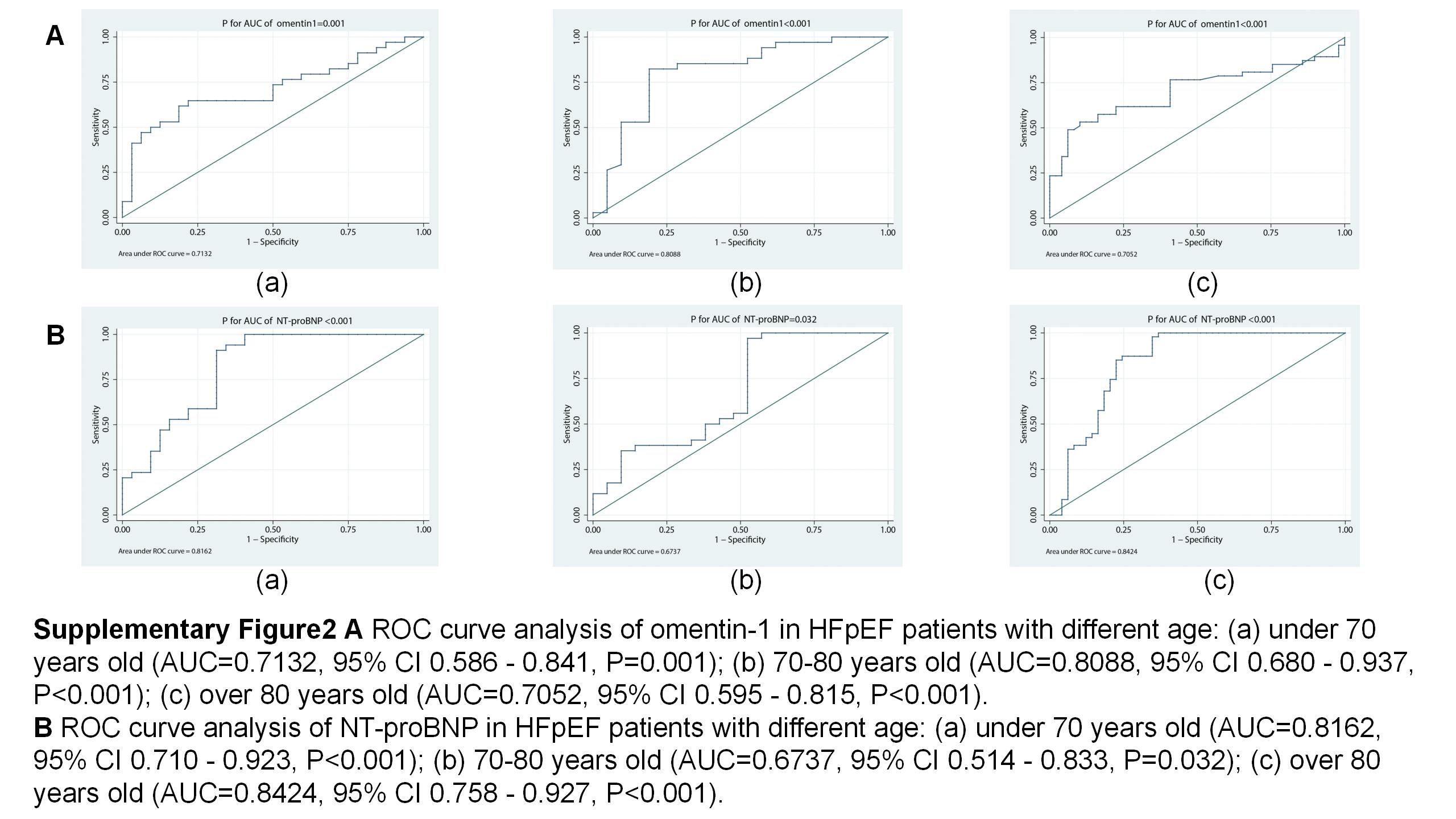

Supplement: Supplementary file 2 — Supporting information. [file CLC-47-e24181-s001.jpg]
